# Subword symmetry in natural languages

**DOI:** 10.1098/rsos.250295

**Published:** 2025-08-21

**Authors:** Olga Pelloni, Rob van der Goot, Peter Ranacher, Ivan Vulic, Tanja Samardzic

**Affiliations:** ^1^ URPP Language and Space, University of Zurich, Zurich, Switzerland; ^2^ Department of Computer Science, IT University of Copenhagen, Copenhagen, Denmark; ^3^ Language Technology Lab, University of Cambridge, Cambridge, UK

**Keywords:** symmetry, subword segmentation, natural language

## Abstract

Symmetric patterns are found in the orderly arrangements of natural structures, from proteins to the symmetry in animals’ bodies. Symmetric structures are more stable and easier to describe and compress, which is why they may have been preferred as building blocks in natural selection. The idea that natural languages undergo an evolutionary process akin to the evolution of species has been pervasive in the study of language. This process might result in symmetric patterns as in other natural structures, but the notion of symmetry is rarely associated with the study of natural language. In this study, we look for symmetric patterns in text data, considering the length of subword units under a range of possible subword analyses. We study the length of subword units in 32 languages and discover that the splits of long words tend to be symmetric regardless of the segmentation method and that some automatic methods give symmetric splits at all word lengths. These results include natural language in the set of phenomena that can be described in terms of symmetry, opening a new research avenue for the empirical study of text data as a structure comparable to various other structures in the natural world.

## 1. Introduction

In the intricate fabric of the natural world, a recurring motif emerges with notable regularity: the phenomenon of symmetry. We can notice symmetrical patterns all around us, whether in the shape of tree leaves, snowflakes, or in our own bodies. Intuitively, symmetry evokes a sense of harmony and order characterized by proportionate and balanced arrangements, such as the two halves of a maple leaf.^1^ In mathematical terms, symmetry is defined as an object’s property that remains invariant under certain transformations [2]. As the dimensions of an object increase, more transformations and symmetry types become possible. For instance, a one-dimensional object can undergo translation (shifting in space) and reflection, while rotation and glide reflection are also possible in a two-dimensional space.

Symmetric patterns are found in the orderly arrangements in crystals, where atoms form repetitive and symmetric groups within a crystal lattice. They are also common in many other structures, from proteins and RNA to the bilateral symmetry found in animals’ bodies. The evolution of symmetry in the morphology of crystals and organisms is explained by the optimization of their functioning. In the case of crystals, the symmetric connections of molecules allow more stability and correspond to a lower energy state [3]. In organisms, bilateral symmetry makes locomotion in one direction more efficient [4]. Symmetric shapes are easy to describe and compress, suggesting that they may have been preferred as building blocks of the genetic code during natural selection [5]. Regular patterns can be observed not only in the morphology of organisms but also in their communicative means. For example, trains of electrical spikes in fungi were found to be highly symmetric [6].

Natural language is sometimes seen as a product of natural selection and evolution [7–9], a perspective dating back to the nineteenth-century positivist view of languages as living beings [10]. The idea that languages undergo an evolutionary process akin to the evolution of species remains prominent to the present day. These processes might result in symmetric patterns, as in other natural structures, but the notion of symmetry is rarely associated with the study of natural language.

To look for possible symmetric patterns in natural language, it is useful to have in mind the distinction between underlying rules (structures) and observable manifestations of language, whether in the form of speech or text, similar to the distinction between genotypes and phenotypes in biology. Focusing on speech and text as the observable manifestation of language, we can spot some regularities that might be symmetric.

Text can be represented as a self-similar lexicographic tree [11, pp. 239–244], which would be a case of scale symmetry. The constant information rate in spoken language [12,13] and in text data [14,15] can also be seen as a kind of symmetry. Some empirical evidence has been found in support of this idea, but the question of whether such symmetries exist in natural language is still open. The constant information rate would be inconsistent with other analyses of text data from the point of view of information theory [16], and the initial findings could not be reproduced [17].

In this study, we look for symmetric patterns in text data from a different point of view. We consider the length of subword units under a range of possible subword analyses. For example, these are some of the possible analyses of four English words:

**Table:** 

plausible	:	plaus ible	plaus i ble	plausi bl e	pl aus ible
credible	:	cred ible	cred i ble	credi bl e	credible
probable	:	prob able	prob a ble	proba bl e	prob able
arguable	:	argue able	argue a ble	argua bl e	arg uable

The first three splits are all correct linguistic analyses at different levels of granularity, while the last column shows an automatic word split called *subword tokenization*.

Although incorrect as linguistic analysis, subword tokenization has recently gained practical value in large language models. Taking subword segments as processing units (instead of whole words) was shown to improve neural machine translation models [18], and subword tokenization has become a standard preprocessing step in training transformers-based [19] models. Automatic tokenizers typically do not reproduce manual analyses, but they seem to find subword segments that are generally helpful in language modelling.

Looking at the length of the subword units, we can notice that some word splits are more balanced than others, regardless of whether they are linguistically plausible or not. We focus on this property and study the length of subword units under varied subword analyses in 32 languages.

Our study is the first to address the presence of symmetric patterns in the length of subword units formed both manually and automatically. We show that the splits of long words tend to be symmetric regardless of the segmentation method and that some automatic methods give symmetric splits at all word lengths. These results include natural language in the set of phenomena that can be described and studied in terms of symmetry, opening a new research avenue for the empirical study of text data as a structure comparable with various other structures in the natural world.

## 2. Related work

In terms of *information theory* applied to natural language, the length of text segments is optimized for efficient communication so that more frequent messages (words) are assigned shorter codes (sequences of sounds or written characters). This regularity is known as the *Zipf’s law of abbreviation* [20], and it has been shown to hold in all studied languages [21]. Longer segments are expected to carry higher average information content [22] than shorter ones. Word length can be approximately predicted from its text frequency [23,24], but the effect varies across languages. The *surprisal* value in a given context has been proposed as a better predictor of word length [22], but, surprisingly, raw frequency turns out to give better predictions [25].

While the relation between word length and frequency has been extensively studied and modelled, the length of subword units has been rather neglected. The tendency of longer higher-level units to be composed of shorter lower-level units (‘the bigger the whole, the smaller the parts’) has been known as the *Menzerath–Altmann’s law* [26,27]. It has been shown to hold for the structure of words [28] and clauses [29], but its role in achieving code efficiency is still to be clarified.

The length of text units is precisely what is manipulated in automatic subword tokenization. No matter how words are split, their parts will become shorter units whose frequency will increase. This leads to shorter vocabularies and fewer rare units. In multilingual processing, smaller units increase the overlap between different languages so that some representations can be shared.

Although we know that shorter (subword) tokens are better units than words for text processing with deep neural networks, the question of how long the subword units should be remains open. The impact of subword tokenization is most commonly assessed in the context of language modelling [30–32] and neural machine translation (NMT) [33–41]. It is sometimes studied in relation to linguistic analyses (syntax, part-of-speech tagging, morphology and semantics) [42,43]. Some tokenizers are regarded as more capable of producing linguistically motivated word splits than others, but there is currently no agreement on whether linguistically plausible splits give better results in the final processing output. Some authors claim that linguistically motivated tokenization improves processing [30,31,37,39,41], while others report that better results are obtained with general compression algorithms [36]. Several papers conclude that there is no significant difference [34] and that there is no general recipe for subword tokenization that suits all languages and language pairs [32,38,40], practical language processing tasks [43] and data size [35].

Before subword tokenization became part of the standard text processing pipeline, methods for segmenting words into smaller units were studied independently of other processing tasks and evaluated directly against a manually created gold standard or on a downstream task. This work has been referred to as *morphological segmentation*. Most of the evaluations have been performed as part of the Morpho Challenge [44], which provided gold standard data for several languages (Finnish, Turkish, English, German, Arabic). Unsupervised algorithms have a long tradition in this domain [45]. Although supervised models [46–48] and finite-state technology [49–51] can give better results in terms of direct evaluation, unsupervised methods are much simpler to build and, without a clear motivation to find the correct segmentation, they prevailed in modern tokenizers.

Measuring text redundancy under different possible subword splits has been proposed as a potential direction toward a more principled criterion for identifying subword units in the unsupervised framework [52]. The units that minimize text redundancy tend to be short, even though, theoretically, they could be long too. Interestingly, the text entropy of many languages converges to almost the same value with such units, and their further analyses can identify language types [53].

The presence of symmetry in the resulting subword units has not been discussed in previous studies despite its relevance to both the scientific and the technological discussion on the structure of text data. Our study is intended to clarify whether the arrangements of subword units obtained by various methods (manual and automatic) are symmetric, introducing a general scientific perspective in the study of natural language and text data.

## 3. Methods

**Figure 1.**
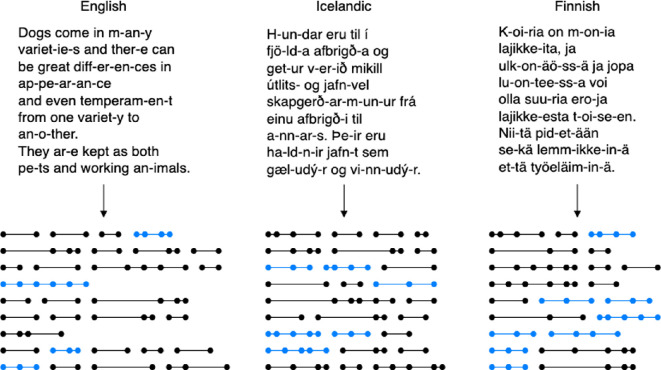
Examples of abstract representations of text samples. Each line represents a word, and the points on the lines represent the boundaries between subword units. The length of the lines is proportional to the length of the corresponding text units. Blue patterns represent more balanced arrangements (symmetric in a broad sense).

The central component of our approach is an abstract representation of text samples, as shown in figure 1. Considering only the length of each subword segment as a measurable property of the subword structure, we aim to find out how the lengths of subword segments are distributed. Do words consist of one long and many short units, or do all units tend to be of similar length? Are lengths of subword units arranged in a *symmetric* way or not?

Given the uncertainty (no consensus) of what subword units exist in reality [54–56], we perform tests under a range of possible splits of words into smaller units considering surface-level manual analyses and several automatic methods developed for the purpose of subword tokenization. We refer to both manual and automatic segmentation with the term *subword segmentation* or *subword splits*. We work with text samples from 32 diverse languages (13 low-resource and 19 high-resource).

### 3.1. Data

The data for all our experiments are texts segmented into subword units both manually and automatically. Since both kinds of segmentation are not available in many languages, we perform our study in two parts. In the first part, we make a direct comparison between manual and automatic word splits on the *Aalto* data, which are available for only three languages. In the second part, we extend the analysis to the *TeDDi* sample, which contains more languages but without the possibility of obtaining both manual and automatic splits for the same languages.

#### 3.1.1. Aalto

The Aalto Morpho Challenge project^2^ provides relatively large datasets where subword units are annotated manually and large unsegmented texts are available for training algorithms for English, Finnish and Turkish. There are more than 1500 manually segmented word types in each language, and the size of the unsegmented text samples is measured in millions of word-level tokens (see more details in appendix A). This resource thus makes it possible to obtain both human and automatic segmentation for the same word type, but it only contains three languages.

#### 3.1.2. TeDDi

For a larger and more representative sample of languages, we extract data from the TeDDi sample [57], which covers almost all languages included in the WALS 100-language sample^3^ and obtains the highest diversity score compared with other multilingual datasets [58]. The details of data extraction can be found in appendix B. In the TeDDi setting, we do not have both manual and automatic segmentation for the same languages. The main sources of manual subword analyses are glossed examples in less studied languages.^4^ The less studied languages also tend to be low-resource languages, where large texts are not available for training automatic tokenizers. The opposite is true for high-resource languages, for which we easily find large texts, but surface-level manual segmentation is rarely available. Language-specific morphological analysers might be used to produce segmentation that follows closely linguistic rules (e.g. [59,60]), but such analysers are hard to use in multilingual settings and not available for most of the TeDDi languages. This means that, in the TeDDi setting, manual segmentation is available only in low-resource languages, while automatic segmentation is available only in high-resource languages. We thus extract manual splits from 13 low-resource languages (Bagirmi, Burushaski, Dani, Imonda, Kayardild, Lavukaleve, Makah, Martuthunira, Maybrat, Ngiyambaa, Piraha, Rama, Tiwi) and automatic splits from 19 high-resource languages (Basque, English, Finnish, French, German, Greek, Hebrew, Hindi, Indonesian, Japanese, Korean, Mandarin, Persian, Russian, Spanish, Tagalog, Thai, Turkish, Vietnamese). These two sets of languages are disjoint, but they still allow us to see whether the patterns observed on the Aalto data hold on a larger sample of languages.

### 3.2. Subword tokenizers

**Figure 2.**
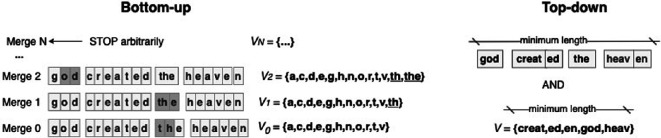
Bottom-up versus top-down subword segmentation. Each box in the text represents a subword unit and the set V contains all the created subword units (members of the vocabulary). The bottom-up algorithms create subword units by merging iteratively pairs of symbols starting from Unicode characters. Top-down methods minimize both the vocabulary and the text length.

Typically, we distinguish between bottom-up compression algorithms (byte pair encoding (BPE) [18] and WordPiece [61,62]) and top-down probabilistic models (Morfessor [63] and the SentencePiece Unigram model [64]). Figure 2 illustrates the main ideas behind these two types of automatic subword segmentation approaches. The bottom-up algorithms start from a text representation where each Unicode character is considered to be a token. They obtain longer subword units by iterative merging of pairs of adjacent symbols, one pair per iteration. BPE merges simply a pair of the two most frequent symbols in a given text sample, while WordPiece merges the pair whose merge increases the most the log-probability of the whole data (the text sample). The process of merging goes on until an arbitrary stop, which is the main hyperparameter to be set. There are currently no universal methods for deciding when to stop. For instance, one can choose 10K merges for an English dataset after trying 1K, 10K and 100K, manually examining the resulting segments and judging that ‘10K gave the most plausible segmentation’ [32, p. 3]. In the top-down approach, all possible subword splits are considered and those splits that maximize the log-probability of the text (globally) are selected. To achieve this objective, top-down models need to optimize the length of the vocabulary and the length of the sequence: short units give a short vocabulary where units have high probabilities, but this makes the sequence long, which decreases the log-likelihood of the data. In theory, the optimal units are those that minimize both vocabulary size and the length of the sequence, but in practice, other values are often found to correspond better to the manual analysis.

For our experiments, we create automatic word splits with four tokenizers, two commonly used ones in the bottom-up category (settings BPE-V and WP) and two from the category of top-down solutions (SPM and Morfessor). Although some hybrid approaches also exist [34,36,40], we limit our study to the main two types which are most widely used. For these methods, we set the hyperparameters in the same way as in previous approaches. The resulting subword units thus generally correspond to those evaluated in previous work. In addition to the commonly used automatic methods, we consider a third bottom-up setting (BPE-MR). All the tokenizers are trained on the data described above (unsegmented texts are used for training), and the resulting vocabularies are applied to obtain automatically segmented texts.

#### 3.2.1. BPE-MR

Our first setting, *BPE Minimum Redundancy*, is intended for exploring the linguistic units obtained with the BPE algorithm stopped at the point where text redundancy reaches the minimum value. The term *redundancy* refers here to an information-theoretic measure that shows how repetitive sequences are. At the starting point of BPE, where each character is a token (Merge 0 in figure 2), text data are extremely repetitive: there are relatively few symbols and each of them occurs very frequently. Text redundancy is reduced by merging the most frequent (repetitive) pairs of symbols because the repetition of the involved characters is reduced. For example, both *t* and *h* appear twice in Merge 0, while each of them appears only once as an individual token in Merge 1. After a number of merges, text redundancy reaches a minimum and starts growing again with further merges [52]. This usually happens after relatively few merges (e.g. 200 in alphabetic, 600−1000 in syllabic scripts), leaving many single characters. This setting is currently not used in practical text processing, but it is interesting for our study because of its general criterion for identifying subword units as symbols that minimize text redundancy. To obtain subword-level segments, we concatenate all consecutive single characters into single units, for example, *p h il i p p in es* →*ph il ipp in es*. To train and apply the BPE-MR vocabulary, we use the subword-nmt library [18].

#### 3.2.2. BPE-V

In this version of BPE, the stopping criterion is a function of the word-level vocabulary size. The word-level vocabulary is the number of unique words in the text that are to be segmented into subword units. Subword tokenization results in a reduction in vocabulary size (subword vocabulary size is smaller than word-level vocabulary size). The number of BPE merges is determined as 0.4×|V|, where |V| is the size of the word-level vocabulary in a raw text (training data). This threshold has been shown to maximize the performance of trained language models on a sample of 64 languages [65] and also used in subsequent work [30]. With this many merges, applying the resulting vocabulary does not leave many adjacent single characters. For training and applying the algorithm in this case, we use the subword-nmt library too.

#### 3.2.3. WordPiece

We train the WordPiece (WP) tokenizer [61], using the implementation from the HuggingFace tokenizers library.^5^ We set a subword vocabulary size parameter for training the same as in the results of the BPE-V setting in order to make the results of the two bottom-up algorithms comparable (see the details on subword vocabulary size in appendix C).

#### 3.2.4. SentencePiece model

We train and apply the SentencePiece unigram model (SPM) [64] using the original SentencePiece tokenizer.^6^ We set the vocabulary size parameter the same as the resulting vocabulary size after training the Morfessor Baseline model, with several changes in languages in which the vocabulary had to be smaller due to the SPM implementation (see the details in appendix C). Following the recommendations in the documentation, we adjust the character coverage parameter for the languages with a large character set: 0.9995 for Japanese and Mandarin, 0.9998 for Korean. The rest of the languages are trained with the character coverage parameter set to 1.

#### 3.2.5. Morfessor

We train our Morfessor model using the Morfessor 2.0 library [66]. Unlike bottom-up tokenizers, Morfessor does not require a stopping criterion (it stops when it reaches its objective), but it can be tuned to give more or less weight to some model parameters, leading to a smaller or larger vocabulary. Following previous work, we use the default hyperparameter settings (without tuning) and, as mentioned above, set the SPM vocabulary size parameter to match the one produced by the default Morfessor settings.

#### 3.2.6. Controlling the vocabulary size

Vocabulary size is the size of the set of all subword units obtained by a segmentation method. When given as a hyperparameter to a tokenizer, it can greatly influence the output of subword tokenization. To control for this factor, we keep the vocabulary size constant in some settings. As mentioned above, we set the size to be the same in BPE-V and WordPiece as two bottom-up algorithms. We also set the SPM vocabulary size to be the same as the one produced with the default settings of Morfessor. Note that despite the set vocabulary size parameter, SPM determines a maximum vocabulary size prior to training. In the case of four languages, this limit was lower than the vocabulary size produced by the default Morfessor on the same input data; thus, we adjusted the vocabulary size parameter accordingly. The vocabulary sizes of BPE-MR and manual segmentation are not arbitrary. The detailed statistics are given in appendix C.

#### 3.2.7. Random-split baselines

To understand better the impact of the vocabulary size on the comparability of different methods, we create a random-split baseline for each setting. For this, we take the number of splits performed by each method (*N*). For example, all inside points on the lines in figure 1 are the splits that are counted: 20 splits in English, 27 splits in Icelandic and 29 splits in Finnish. We then perform the same number of splits randomly. To select random splits, we take the positions of all characters in a given text (except the word boundaries) as the range of integers from which we make *N* random draws. We then insert a split after each drawn position. For each random-split baseline, we repeat this procedure five times with different random seeds.

### 3.3. Analyses

**Figure 3.**
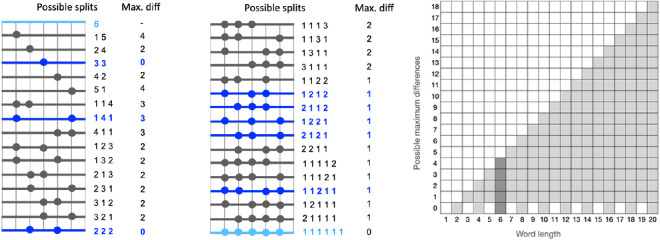
The space of possible values used in the analyses. Left: all 32 possible subword splits for a word of length 6 (an example length) with the associated maximum differences; the maximum difference is the difference in length between the longest and the shortest subword unit in a given split; blue patterns are symmetric, light blue excluded from all counts. Right: all possible values of the maximum difference in length of subword units for word lengths 1 to 20; note that possible differences for the example word length 6 are 0, 1, 2, 3, 4.

To describe the arrangements of the subword unit lengths obtained with different segmentation methods, we consider patterns in a one-dimensional space (shown as segmented lines in figure 1). We measure the length of each subword unit, taking Unicode characters to be the units of length so that each character has a length of 1. Since this is a one-dimensional space, we can have *translational symmetry*, where the same sequence of lengths is repeated within the same word, and *reflectional symmetry*, where a sequence of lengths is reversed with respect to the centre of a given word. For example, the length patterns (3, 3), (2, 2, 2) and (1, 2, 1, 2) in figure 3 are cases of translational symmetry. The patterns (1, 4, 1), (2, 1, 1, 2) and (1, 2, 2, 1) are cases of reflectional symmetry.

This measurement is best suited for alphabetic scripts, where most distinctions can be made, but it is applicable to all other scripts as well. The observed differences in non-alphabetic scripts will be smaller (due to shorter word length), but the geometrical patterns are expected to be invariant. Note also that a great majority of languages (85% in the WALS 100L sample) are written in alphabetic scripts.

To take into account the fact that the combinatorial space of all possible arrangements of lengths changes with the length of a word, we group the measurements according to the word length. The second factor that impacts the arrangements is the choice of the subword segmentation method. We thus make observations about the arrangements of subword segments’ length for each word length and segmentation method separately. We perform two kinds of analyses, each approximating the notion of symmetry from a different point of view.

#### 3.3.1. Analysis 1: the proportion of symmetric patterns

In the first analysis, we measure the difference $D_{l}$ between the *observed* $O_{l}$ and the *expected* $E_{l}$ proportion of symmetric splits in all the words of the same length l.


(3.1) $$D_{l}=\frac{O_{l}-E_{l}}{E_{l}}$$

Since each word length has a different expected proportion (as explained below), we normalize the difference between the observed and the expected proportion by dividing each difference by the corresponding expected value. In this way, the differences across different word lengths become comparable. We repeat the calculation for each subword segmentation method and its corresponding random-split baseline.

The expected proportion is the ratio between the size of the set of possible symmetric splits $S_{l}$ and the total of all possible splits $T_{l}$ for a given word length l.


(3.2) $$E_{l}=\frac{|S_{l}|}{|T_{l}|}$$

To find the observed proportion, we count the instances of symmetric splits $S_{l}$ in all the word tokens of the length l in the segmented text samples in all languages and divide this number by the total number of word tokens N of the length l.


(3.3) $$O_{l}=\frac{count(S_{l})}{N_{l}}$$

For example, figure 3 shows all possible patterns for all words of length 6. To find observed proportions of symmetric patterns, we count all instances of words of length 6 in all languages whose segmentation corresponds to any of the dark blue patterns. We divide this number by the total number of words of length 6 in all languages. To exclude the edge cases (the patterns in light blue), we filter out all the words where the number of subword segments is 1 and also those where the number of subword segments is equal to the length of the word (the latter typically does not happen in real splits). For calculating the expected proportion in this case, we take the number of dark blue patterns and divide it by the number of all possible splits excluding the edge cases, which gives $\frac{8}{30}$.

We use the cardinality sign in (3.2) to signal that the expected proportions are estimated at the level of the pattern type while the observed proportions in (3.3) are calculated over instances of patterns. The number of possible splits grows exponentially with the word length ($|T_{l}|=2^{l-1}$). The maximal number of symmetric splits also grows as a function of word length, but more slowly. In the case of reflectional symmetry $|S_{l}|=2^{ceiling(\frac{l-1}{2})}$. The function is more complex in the case of translational symmetry because the space of possibilities depends on the length parity of subword units in a more complex way. For simplicity, we find the latter number by enumeration. Taking into account both types of symmetry, the expected proportions of symmetric splits drop with the increasing word length, which is taken into account by normalization in (3.1).

#### 3.3.2. Analysis 2: subword evenness

**Figure 4.**
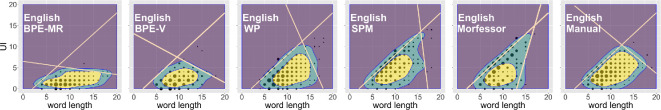
Examples of calculating the evenness score in Analysis 2. Yellow: the KDE density area level 1; blue: the KDE density level 2; violet: KDE density area level 3 (no density). The SuE score is the angle between the two lines that approximate the boundary of the density area. The size of the points represents the frequency of each observed difference, but this information is not taken into account when calculating SuE.

In the second analysis, we consider a view of symmetry that is more flexible than the strict view taken in the first analysis. Here, we focus on the patterns where all the subword units are of similar length. We distinguish between *even* and *uneven* patterns. For example, the most even splits in figure 3 are (3, 3) and (2, 2, 2). The most uneven splits are (1, 5) and (5, 1). The degree of evenness can be expressed as the difference in length between the shortest and the longest subword segment (Max. diff in figure 3).^7^ It is bound by the word length as shown on the right-hand side in figure 3. For example, possible values for length 6 are 0, 1, 2, 3, 4. Note that, unlike the strict notion of symmetry in the first analysis, the notion of evenness is gradual. If the observed scores tend to group around the ‘diagonal’, then the observed patterns are uneven (while they can be symmetric or not). Grouping of the patterns along the *x*-axis would indicate a strong preference for even splits (while some of these patterns would not be symmetric in the strict sense). As a measure of evenness, we calculate the subword evenness (SuE) score [67], which summarizes the trends in the distribution of maximal differences in the data. In particular, the SuE measure shows how the space of possible scores (the right-bottom triangle on the right side of figure 3) is populated in real observations. The core of this measure is a kernel density estimation (KDE) over the observed values: the closer the density area to the *x*-axis, the higher the SuE value. Since the SuE measure already takes into account word length, a single score applies to all word lengths. Like in the previous analysis, we calculate one score for each subword segmentation method and its corresponding random-split baseline. Figure 4 shows an example plot for each subword segmentation method summarizing the distribution of maximal differences in English data.

## 4. Results

For each of our findings, we first present the results obtained on the Aalto dataset, on which we can make direct comparisons between manual and automatic subword segmentation methods. We then show the same findings across a larger set of maximally diverse languages in the TeDDi sample, for which we make indirect comparisons and where the data come from various sources. Despite the fully disjoint sets of languages and a rather small number of tokens in the manually segmented data, the trends observed on the Aalto dataset are confirmed on the TeDDi data.

### 4.1. Overall more symmetry in long words

**Figure 5.**
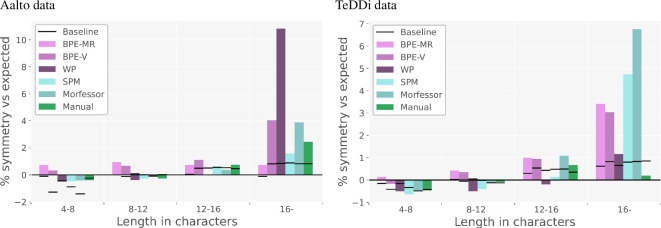
Results of Analysis 1: the difference between the observed and expected proportion of symmetric splits. Positive values represent more symmetry than expected, negative less than expected. Word lengths on the *x*-axis are grouped into four bins. All the reported values are bin averages for each method. The random-split baseline values are averaged over five random seeds.

Figure 5 shows that the proportion of symmetric splits grows with the word length in all subword segmentation settings, both manual and automatic. In the first two bins (word length up to 12), the observed values are below and close to the expected values in some settings, while they are increasingly above the expected values for all settings at word lengths above 12.

When compared with random-split baselines, the observed proportions tend to be higher, with the difference mostly increasing with word length. The cases where the observed proportions are below their corresponding random-split baselines are mostly in the second bin (word lengths 8−12), where the values are generally close to the expected ones. In some methods (BPE-MR, BPE-V), the observed proportions are always above the random-split baselines, as described in more detail below. The fact that the random-split proportions follow the pattern of symmetry growing with word length can be explained by the constraints put on the vocabulary size. Recall that we aimed at making random-split baselines as comparable as possible to each subword segmentation setting by controlling the vocabulary size. We created a separate set of random subword splits for each method so that the vocabulary size is the same in a given method and its corresponding random-split baseline. As a consequence, each random baseline contains only a subset of possible splits limited by the vocabulary size in a given setting. This is probably the reason why random-split proportions depart from the expected values, mostly following the trend in the observed (real) data. Note, however, that the random-split baseline values are rather similar within a word length bin. While different subword segmenting methods will result in varied vocabulary sizes, the variation in random-split values is much smaller than in the real subword segmentation methods. This observation points to a rather limited impact of the vocabulary size on the proportion of symmetric splits.

The same trend comes out of the second analysis as well, where even patterns are observed more in longer words. We can see in figure 4 that the variation in the shape of the KDE density area is determined by the presence or absence of uneven splits in long words (lengths above 10). This figure shows only the distributions for English, but it illustrates the trends that we find in all other languages as well. More even splits in longer words result in a more obtuse angle between the two lines (higher SuE score), while the presence of uneven splits results in a sharper angle (lower SuE score). The shapes of the density areas differ to some degree depending on the subword segmentation method. Manual segmentation patterns with BPE-MR in well-defined density regions (large yellow, small blue areas), while relatively big blue areas in the other settings, especially in SPM and Morfessor, indicate poor density, i.e. little consistency. On the other hand, the high-density (yellow) areas tend to be relatively close to the *x*-axis in settings, indicating a preference towards even units.

### 4.2. More symmetry in bottom-up than in top-down tokenizers

**Figure 6.**
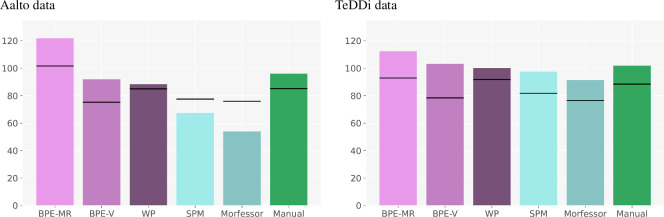
Results of Analysis 2: the distribution of the SuE scores. On the left: the Aalto dataset, segmented by five algorithms and segmented manually. All the reported values are averages for each segmentation method. The horizontal line represents the random-split baseline values averaged over five random seeds for each segmentation method.

Comparing the subword segmentation methods in both figures 5 and 6, we see a consistent difference between bottom-up (BPE-MR, BPE-V) and top-down (SPM, Morfessor) tokenizers. This difference is especially interesting knowing that both BPE-MR and BPE-V are naive in terms of statistical modelling because they use only raw frequency for finding subword units (merging two most frequent adjacent pairs of symbols), while both top-down algorithms employ more sophisticated statistical modelling (maximizing the log-probability of the whole text sample).

Figure 5 shows that the two bottom-up tokenizers produce symmetric splits at all word lengths (with one exception of BPE-V on the TeDDi data), while the two top-down algorithms get above the expected values only in long words. Note that this difference cannot be attributed to the difference in the vocabulary size because the random-split baselines are not affected by the differences in the vocabulary size. Figure 6 shows that bottom-up tokenizers also give more even splits. This figure shows the average SuE scores per setting with the corresponding random-split baselines (per-language scores are given in appendix E). In the case of Aalto data, the SuE values of BPE-MR and BPE-V are above the corresponding random-split baselines, while those of SPM and Morfessor are below the random-split baselines. In the case of TeDDi data, all the scores are above the random-split baselines, and the differences between the methods are smaller, but the ranking remains almost identical, confirming once again the robustness of the difference between the two types of tokenizers. While BPE-MR and BPE-V behave in a very similar way across the settings, BPE-MR results in the most symmetric and most even subword units overall. This finding is important for relating our results to previous work, and we will return to it in the discussion.

The criterion used for merging characters into subword units in bottom-up automatic methods seems to impact the symmetry of resulting subword unit lengths. WP is a bottom-up algorithm like BPE-MR and BPE-V, but it is similar to the top-down algorithms in using the probability of the symbols as a criterion for merging: instead of merging the most frequent pair of symbols, it merges the pair that increases the likelihood of the text sample (the sequence of tokens) the most. This will be the pair of symbols with the strongest association (symbols co-occurring together most of the time and rarely one without the other). As a result, WP will tend to merge pairs of rare symbols that happen to be adjacent when they occur. This approach results in unstable WP values and bigger differences between the experimental settings.

**Figure 7.**
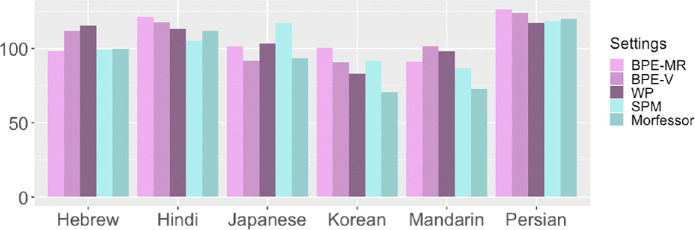
SuE values in six languages with non-alphabetic scripts in the TeDDi sample, only automatic tokenization settings.

A limitation of using Unicode characters as units of length is their varied mapping to the sounds. Depending on the type of the script, characters can stand for single sounds (alphabetic), consonants (abjad), syllables (syllabic) or bigger meaningful units (logographic). The mapping is further complicated by the canonical decomposition (normalization form D, NFD)^8^ in some implementations. The TeDDi dataset includes various scripts, which allows us to check whether the difference between the two types of tokenizers is preserved in non-alphabetic scripts. In figure 7, we plot the SuE values for several languages that use non-alphabetic scripts (more details in appendices D and E). In some cases, WP or BPE-V values are higher than in BPE-MR (Hebrew, Mandarin); in others, SPM reaches the values typical for bottom-up algorithms (Japanese, Korean). These are the cases that might require some further investigation. For now, we note that the main pattern that holds in the full dataset is preserved in the subset of languages with non-alphabetic scripts: bottom-up algorithms give more even splits than top-down ones.

### 4.3. Manual versus automatic subword units

The results are most surprising in the manual subword segmentation setting, where the proportion of symmetric splits stays relatively close to the expected values and to the random-split baselines compared with the automatic methods. In the evenness analysis, manual splits tend to be between the two kinds of automatic methods and also relatively close to the random-split baseline. Given the results of the two analyses, we can say that manually created subword units tend to be more even than symmetric in the narrow sense. While top-down automatic methods are usually regarded as more linguistically motivated, it turns out that they pattern with the manual splits only in the strict sense of symmetry. In the evenness analysis, the results of the bottom-up methods are more similar to those in manual subword segmentation.

**Figure 8.**
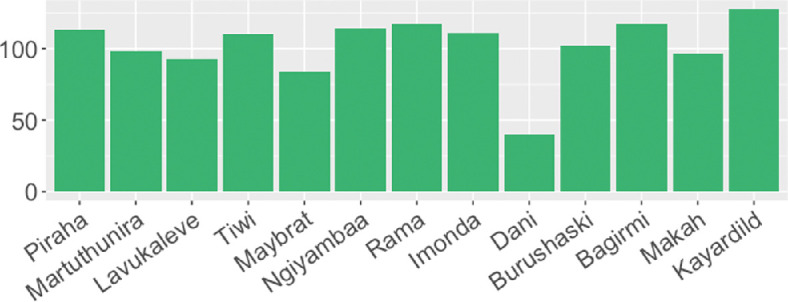
SuE values in 13 low-resource languages (only manual subword splits) in the TeDDi sample, sorted by the data size in decreasing order.

It is especially surprising that very similar trends are observed in the evenness analysis in the Aalto and TeDDi data, despite the fact that manually segmented texts in the TeDDi data can be very short and noisy due to non-standardized writing and multiple sources (the details about the data size are available in appendix B). Zooming in the TeDDi sample, figure 8 shows rather similar SuE values across languages, with only one outlier (Dani). On the other hand, the relatively low proportion of symmetric splits in the longest words in figure 5 could be attributed to the small data size. Since long words are relatively rare and manually segmented texts in the TeDDi sample are very short, observed proportions of symmetric patterns in manual splits sometimes rely on only several words. Any overlooked boundary might have a big impact on the observed proportions in this setting. The SuE score seems more robust to small and noisy datasets than a strict measure of symmetry. However, the question of whether the proportion of symmetric patterns would be similar to the Aalto value with more larger texts in the TeDDi data remains open.

## 5. Discussion

Our findings suggest that natural language can be described in terms of symmetry like the morphology of different physical objects in natural science or more abstract objects and processes in psychology, cognitive sciences and artificial intelligence. Previous studies of perceptual organization in gestalt psychology, for instance, named symmetry as one of the key principles involved in the perceptual grouping of objects. The human mind will tend to perceive a visual field consisting of multiple objects as a single figure if the shape of the visual field is symmetrical [68]. The speed of visual processing is also shown to be impacted by symmetry: symmetric shapes are processed faster than asymmetric ones, and vertically symmetric shapes, in particular, are preferred starting from infancy [69]. The perception principles known for human cognition have found application in artificial intelligence as well, for example, for building AI agents that use symmetry as the organizing principle for enhanced visual reasoning [70]. Here, we discuss the broader implications of our findings for the theoretical study of natural language and for its computational modelling.

### 5.1. Subword symmetry and frequency effects

The fact that natural language functions as an efficient communication code means that shorter words are more frequent. Shorter words are also those where we find little symmetry. A possible explanation for this finding is that frequency of use diminishes symmetry in the arrangements of subword units. For instance, irregular past tense in English is more likely in frequent verbs such as ‘keep, kept’ than in less frequent verbs such as ‘preserve, preserved’. Less frequent words are more regular and can be analysed into subword units. The opposition between regularity and frequency is widely discussed in the linguistic literature, especially in the context of the productivity of subword units [71]. More productive patterns of formation in less frequent words are more analysable and compositional, while frequent words tend to be formed in an unproductive way (less analysable and compositional). It has also been shown that frequent words are more susceptible to regular phonetic changes. For instance, final *-t* and *-d* in words such as ‘just’, ‘perfect’, ‘child’ or ‘grand’ tend to be omitted in American English, but this happens more in frequent than in rare words [72]. Such contractions of forms are interpreted as one of the frequency effects that impact the structure of language. Other frequency effects are discussed too [73], but more from the point of view of grammaticalization, which is not directly related to our topic.

Although the studies in productivity usually do not deal with the length of words, we can attribute the productivity properties to longer words on the basis of information theory (see §2). Our findings are in line with the opposition between frequency and regularity because we find symmetric patterns only in long words. We can conclude that subword units can be described as symmetric when they are transparent, but they are not transparent in shorter words. In addition to providing new empirical evidence for the structural distinction between short (frequent) and long (infrequent) words, our study shows that symmetry starts increasing at a specific (approximate) length that seems constant across languages. It might be possible to relate this turning point to a specific cognitive constraint to gain deeper insights into cognitive processes involved in the use of natural language.

### 5.2. Subword evenness and the information rate in text data

Previous discussion relevant to the notion of symmetry in natural languages was concerned with the distribution of information over word positions in texts. By making more predictable items shorter and giving more room to less predictable (more informative) items, speakers achieve communicative efficiency, possibly trying to keep a relatively constant flow of information, which would optimize the use of the communication channel. If the objective of maintaining a constant flow exists indeed in language use, it is probably in conflict with other (unknown) objectives that prevent its full realization [16].

Our study departs from the idea of probability estimation and introduces a new perspective of text as a one-dimensional geometric object. By studying the length of subword units, we can point to a regularity in the organization of text that is outside of the probability estimation framework. This regularity is what allows BPE-MR to create the most even subword splits. Recall that BPE-MR merges only a few hundred most frequent pairs of symbols, leaving all the rest of the text as single characters. All the adjacent single characters are concatenated into subword units. The fact that the resulting units are even means that the concatenated units are of similar length too. This further means that the most frequent pairs of symbols, whose merging minimizes text redundancy, are rather evenly spaced over text. The repeated (frequent) units thus alternate with new (infrequent) ones in approximately even steps. This might be the subword-level mechanism for distributing the information content in a uniform way across the sequences. The fact that the two naive algorithms find more symmetric and even subword units than those that rely on probability estimation remains puzzling and worth investigating further in future research. The same holds for the manual analysis since some of the units found by BPE-MR could be considered linguistically irrelevant and are thus not treated as structural units. This might be the reason why manual splits are less even than those obtained by BPE methods. Our findings suggest that recurrent subword units might play a role in distributing information despite not being recognized as structural units in manual analyses and in probabilistic automatic subword tokenization.

### 5.3. Implications for language modelling

Theoretical study of natural language and machine learning are viewed as opposite directions in practical language processing [74]. As machine learning is gaining ground thanks to the successes in practical tasks, the popularity of theoretical linguistics is diminishing. With the introduction of deep neural networks, large language models are starting to capture a good deal of how natural language works, but with almost no reliance on the theory of language or any other explicit linguistic knowledge.

Input text segmentation (tokenization) is currently the only processing step that remains outside of deep networks. Many researchers see this as a major problem and would prefer to integrate input segmentation into deep networks, taking sequences of bytes (bytes roughly encode Unicode characters) as input and learning text segmentation together with other levels of representation [75,76]. Up to now, however, the integration efforts have not resulted in clear improvements over separate subword tokenization.

As the tokenization problem persists in text processing, our study offers a scientific basis for developing more principled practical solutions, removing the need to integrate subword tokenization into large language models. The BPE-MR tokenizer, which is currently not in use in practical applications, is a simple method for obtaining the most symmetric and even subword units in any language. If symmetry of subword units is indeed the subword-level mechanism for optimizing the distribution of information in text data, which is still to be shown, symmetric subword tokenization might turn out to be a new basis for dealing with the problem of cross-lingual biases in subword tokenization such as over-segmentation in languages other than English [77], which also causes economic and societal biases [78]. It might prove to be a simpler alternative to current attempts to obtain a more representative tokenization in a multilingual context [79,80]. Rooted in information theory, symmetric tokenization can also help language models distinguish between redundant and informative units and thus extract more information from text. In this scenario, the size of the vocabulary (and thus the length of subword units) is determined by the minimum redundancy criterion and ceases to be an arbitrary hyperparameter, which is necessary for improving the portability of large language models across tasks and languages.

## 6. Conclusion

We have introduced a new perspective in the study of subword units in natural languages manifested as text samples. Regarding text as a one-dimensional object whose main property is length, we show that lengths of subword units tend to be more symmetric in long than in short words. This distinction holds for all subword segmentation methods that we considered, including manual segmentation. A comparison of segmentation methods reveals the impact of the modelling choice on the symmetry of the resulting segments: naive methods that rely on raw frequency produce more symmetric units than probabilistic methods. Manual segmentation turns out relatively symmetric only in the sense of evenness (not strict symmetry). The most symmetric units are produced by an automatic subword segmentation method (BPE-MR) that minimizes text redundancy.

Our findings are in line with the known opposition between frequency and regularity discussed in the linguistic literature and contribute new insights concerning the uniform information density hypothesis. They also provide a scientific basis for dealing with the problem of subword tokenization in large language models, suggesting a simple and principled method for achieving symmetry in subword segmentation in any language.

## Figures and Tables

**Figure 9.**
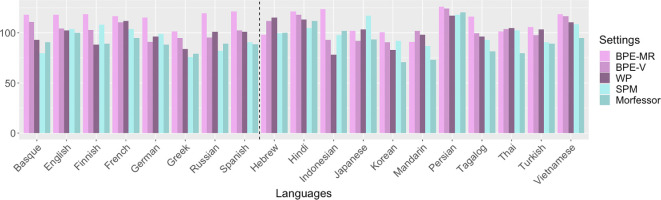
SuE values in19 high-resource languages. On the left: European languages, on the right: non-European languages.

**Figure 10.**
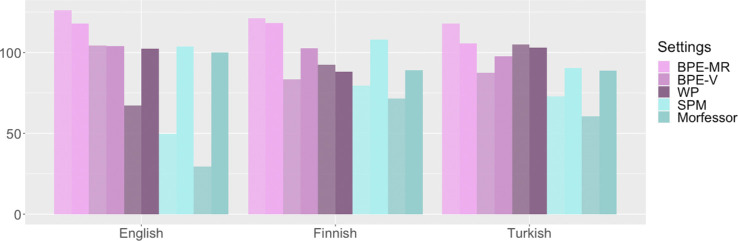
SuE values in English, Finnish and Turkish in the Aalto (more transparent) and the TeDDi (less transparent) datasets. The models seem to depend on the data size more than the other algorithms; however, the general decreasing trend in values remains similar.

## Data Availability

Data and relevant code for this research work are stored in GitHub [81–83] and have been archived within the Zenodo repository [84–86]. The Aalto Morpho Challenge dataset is available on the project page: http://morpho.aalto.fi/events/morphochallenge2010/datasets.shtml.

## Footnotes

^1^
As a reminder, the word ‘symmetry’ originates from Greek symmetros, composed from ‘syn’ (together) and ‘metros’ (measure), which literally means ‘commensurate with’ or ‘having a common measure’ [1].

^2^

http://morpho.aalto.fi/events/morphochallenge2010/

^3^

https://wals.info/languoid/samples/100

^4^

https://www.eva.mpg.de/lingua/resources/glossing-rules.php

^5^

https://huggingface.co/docs/tokenisers/python/latest/

^6^

https://github.com/google/sentencepiece

^7^
This score is similar to the distance from the mean length but simpler to use in further calculations.

^8^

https://unicode.org/reports/tr15/

## Appendix A. Aalto data extraction

From the manually segmented dataset, we take the concatenated lists of training and development sets of the gold standard segmentations, and extract only the segmentations without morphological labels (table 1).

**Table 1.** Number of tokens and types in the dataset.

language	word list (tokens)	word list (types)	gold standard
English	221 715 953	878 036	1686
Finnish	65 734 026	2 928 030	1835
Turkish	12 862 393	617 298	1760

We train the models on the full word lists, where we multiply each word by its frequency, thus reconstructing the original corpora. Once the models are trained, we apply them to the unsegmented version of the gold standard list.

## Appendix B. Languages in the TeDDi data

We make a subset of 19 high-resource languages of 1 000 000 tokens per language. When possible, we balance the genres. For the experiments, we use one-column text files with one word token per line (tables 2–5).

**Table 2.** Data details for the chosen 19 high-resource languages.

language	fiction	other	total
Basque	0	1 000 003	1 000 003
English	500 001	500 003	1 000 004
Finnish	500 008	499 994	1 000 002
French	500 001	499 999	1 000 000
German	500 003	499 997	1 000 000
Greek	500 001	500 006	1 000 007
Hebrew	139 483	860 521	1 000 004
Hindi	0	1 000 000	1 000 000
Indonesian	0	1 000 015	1 000 015
Japanese	500 177	499 825	1 000 002
Korean	0	1 000 013	1 000 013
Mandarin	500 029	499 973	1 000 002
Persian	26 685	973 321	1 000 006
Russian	70 025	929 982	1 000 007
Spanish	500 009	500 012	1 000 021
Tagalog	500 004	500 000	1 000 004
Thai	0	1 000 000	1 000 000
Turkish	0	1 000 000	1 000 000
Vietnamese	0	1 000 003	1 000 003

**Table 3.** Number of word tokens in the low-resource manually segmented data, sorted in decreasing order. The data is taken from the grammars and the fieldwork materials, legally available for use.

language	ISO	tokens
Piraha	myp	4292
Martuthunira	vma	3144
Lavukaleve	lvk	1537
Tiwi	tiw	1201
Maybrat	ayz	737
Ngiyambaa	wyb	691
Rama	rma	401
Imonda	imn	296
Dani (Lower Grand Valley)	dni	229
Burushaski	bsk	203
Bagirmi	bmi	66
Makah	myh	32
Kayardild	gyd	27

**Table 4.** Typological details for the 19 high-resource languages.

language	ISO	genus (WALS)	family (WALS)
Basque	eus	Basque	Basque
English	eng	Germanic	Indo-European
Finnish	fin	Finnic	Uralic
French	fra	Romance	Indo-European
German	deu	Germanic	Indo-European
Greek	ell	Greek	Indo-European
Hebrew	heb	Semitic	Afro-Asiatic
Hindi	hin	Indic	Indo-European
Indonesian	ind	Malayo-Sumbawan	Austronesian
Japanese	jpn	Japanese	Japanese
Korean	kor	Korean	Korean
Mandarin	cmn	Chinese	Sino-Tibetan
Persian	pes	Iranian	Indo-European
Russian	rus	Slavic	Indo-European
Spanish	spa	Romance	Indo-European
Tagalog	tgl	Greater Central Philippine	Austronesian
Thai	tha	Kam-Tai	Tai-Kadai
Turkish	tur	Turkic	Altaic
Vietnamese	vie	Viet-Muong	Austro-Asiatic

**Table 5.** Typological details for the 13 low-resource languages.

language	ISO	family (WALS)	macroarea (WALS)
Bagirmi	bmi	Central Sudanic	Africa
Burushaski	bsk	Burushaski	Eurasia
Dani	dni	Trans-New Guinea	Papunesia
Imonda	imn	Border	Papunesia
Kayardild	gyd	Tangkic	Australia
Lavukaleve	lvk	Solomons East Papuan	Papunesia
Makah	myh	Wakashan	North America
Martuthunira	vma	Pama-Nyungan	Australia
Maybrat	ayz	West Papuan	Papunesia
Ngiyambaa	wyb	Pama-Nyungan	Australia
Piraha	myp	Mura	South America
Rama	rma	Chibchan	North America
Tiwi	tiw	Tiwian	Australia

Fiction texts are taken from the Gutenberg Project, the other texts come from the Parallel Bible Corpus, OpenSubtitles, Universal Declaration of Human Rights.

## Appendix C. Vocabulary size

See (tables 6–8).

**Table 6.** Vocabulary size of the resulting subwords, 19 high-resource languages in the TeDDi dataset in all settings.

language	setting	V
Basque	BPE-MR	4169
Basque	BPE-V	20 470
Basque	WP	20 470
Basque	SPM	24 010
Basque	Morfessor	24 010
English	BPE-MR	4306
English	BPE-V	11 026
English	WP	11 026
English	SPM	18 279
English	Morfessor	18 279
Finnish	BPE-MR	5314
Finnish	BPE-V	33 219
Finnish	WP	33 219
Finnish	SPM	38 988
Finnish	Morfessor	38 988
French	BPE-MR	6018
French	BPE-V	14 595
French	WP	14 595
French	SPM	24 020
French	Morfessor	24 020
German	BPE-MR	7426
German	BPE-V	18 301
German	WP	18 301
German	SPM	23 579
German	Morfessor	23 579
Greek	BPE-MR	13 624
Greek	BPE-V	27 078
Greek	WP	27 078
Greek	SPM	42 587
Greek	Morfessor	42 587
Hebrew	BPE-MR	11 092
Hebrew	BPE-V	19 235
Hebrew	WP	19 235
Hebrew	SPM	31 965
Hebrew	Morfessor	31 965
Hindi	BPE-MR	12 697
Hindi	BPE-V	12 172
Hindi	WP	12 172
Hindi	SPM	19 879
Hindi	Morfessor	19 879
Indonesian	BPE-MR	2945
Indonesian	BPE-V	10 039
Indonesian	WP	10 039
Indonesian	SPM	15 379
Indonesian	Morfessor	15 379
Japanese	BPE-MR	24 965
Japanese	BPE-V	21 704
Japanese	WP	21 704
Japanese	SPM	13 066
Japanese	Morfessor	18 970
Korean	BPE-MR	76 211
Korean	BPE-V	63 164
Korean	WP	63 164
Korean	SPM	51 418
Korean	Morfessor	51 418
Mandarin	BPE-MR	40 432
Mandarin	BPE-V	39 639
Mandarin	WP	39 639
Mandarin	SPM	17 010
Mandarin	Morfessor	26 255
Persian	BPE-MR	12 463
Persian	BPE-V	16 978
Persian	WP	16 978
Persian	SPM	22 249
Persian	Morfessor	22 249
Russian	BPE-MR	7483
Russian	BPE-V	22 209
Russian	WP	22 209
Russian	SPM	32 025
Russian	Morfessor	32 025
Spanish	BPE-MR	4059
Spanish	BPE-V	17 070
Spanish	WP	17 070
Spanish	SPM	25 620
Spanish	Morfessor	25 620
Tagalog	BPE-MR	7835
Tagalog	BPE-V	20 869
Tagalog	WP	20 869
Tagalog	SPM	27 541
Tagalog	Morfessor	27 541
Thai	BPE-MR	10 609
Thai	BPE-V	5149
Thai	WP	5149
Thai	SPM	10 256
Thai	Morfessor	11 242
Turkish	BPE-MR	8021
Turkish	BPE-V	27 285
Turkish	WP	27 285
Turkish	SPM	29 248
Turkish	Morfessor	29 248
Vietnamese	BPE-MR	5856
Vietnamese	BPE-V	5974
Vietnamese	WP	5974
Vietnamese	SPM	5951
Vietnamese	Morfessor	7822

**Table 7.** Vocabulary size of the resulting subwords, Aalto dataset in all settings. Test means the vocabulary size of the gold-list data, the same as in the manual setting. Train means the vocabulary size measured on the training data.

language	setting	V(test)	V(train)
English	BPE-MR	586	61 002
English	BPE-V	1963	323 227
English	WP	1917	323 227
English	SPM	1748	254 507
English	Morfessor	1801	308 519
English	Manual	1676	—
Finnish	BPE-MR	625	75 742
Finnish	BPE-V	2516	1 100 648
Finnish	WP	2877	1 100 648
Finnish	SPM	2789	363 920
Finnish	Morfessor	2522	669 017
Finnish	Manual	2161	—
Turkish	BPE-MR	563	31 491
Turkish	BPE-V	2277	227 564
Turkish	WP	1790	227 564
Turkish	SPM	1976	201 889
Turkish	Morfessor	2044	201 889
Turkish	Manual	1314	—

**Table 8.** Vocabulary size of the resulting subwords, 13 low-resource languages in the TeDDi dataset, manually segmented.

language	V
Bagirmi	480
Burushaski	382
Dani (Lower Grand Valley)	74
Imonda	1094
Kayardild	39
Lavukaleve	222
Makah	185
Martuthunira	751
Maybrat	1611
Ngiyambaa	981
Piraha	176
Rama	51
Tiwi	357

## Appendix D. SuE score: tables of results

See (tables 9–12).

**Table 9.** SuE values in the Aalto dataset, five algorithms and the manual setting.

language	SuE score	setting
English	126.14158	BPE-MR
English	104.50886	BPE-V
English	67.42756	WP
English	49.87653	SPM
English	29.52247	Morfessor
English	93.39345	Manual
Finnish	121.19375	BPE-MR
Finnish	83.44494	BPE-V
Finnish	92.30891	WP
Finnish	79.53355	SPM
Finnish	71.71153	Morfessor
Finnish	102.71497	Manual
Turkish	118.06692	BPE-MR
Turkish	87.56555	BPE-V
Turkish	104.92685	WP
Turkish	72.85846	SPM
Turkish	60.77371	Morfessor
Turkish	91.84246	Manual

**Table 10.** Comparison of the SuE values in English, Finnish, Turkish in both datasets, five algorithms.

language	SuE score	setting	dataset
English	126.14158	BPE-MR	Aalto
English	104.50886	BPE-V	Aalto
English	67.42756	WP	Aalto
English	49.87653	SPM	Aalto
English	29.52247	Morfessor	Aalto
English	118.04372	BPE-MR	TeDDi
English	104.06554	BPE-V	TeDDi
English	102.49587	WP	TeDDi
English	103.64609	SPM	TeDDi
English	99.97943	Morfessor	TeDDi
Finnish	121.19375	BPE-MR	Aalto
Finnish	83.44494	BPE-V	Aalto
Finnish	92.30891	WP	Aalto
Finnish	79.53355	SPM	Aalto
Finnish	71.71153	Morfessor	Aalto
Finnish	118.35456	BPE-MR	TeDDi
Finnish	102.64364	BPE-V	TeDDi
Finnish	88.0279	WP	TeDDi
Finnish	107.97796	SPM	TeDDi
Finnish	89.03298	Morfessor	TeDDi
Turkish	118.06692	BPE-MR	Aalto
Turkish	87.56555	BPE-V	Aalto
Turkish	104.92685	WP	Aalto
Turkish	72.85846	SPM	Aalto
Turkish	60.77371	Morfessor	Aalto
Turkish	105.6798	BPE-MR	TeDDi
Turkish	97.74496	BPE-V	TeDDi
Turkish	103.16006	WP	TeDDi
Turkish	90.41201	SPM	TeDDi
Turkish	88.97014	Morfessor	TeDDi

**Table 11.** SuE values in the TeDDi dataset, 13 low-resource languages, manually segmented.

language	SuE score
Bagirmi	116.86617
Burushaski	101.92105
Dani (LowerGrandValley)	39.79565
Imonda	111.00036
Kayardild	127.35566
Lavukaleve	92.28143
Makah	96.32803
Martuthunira	98.45394
Maybrat	83.95204
Ngiyambaa	113.68764
Piraha	113.34718
Rama	117.08743
Tiwi	110.3288

**Table 12.** SuE values in the TeDDi dataset, 19 high-resource languages, five algorithms.

language	SuE score	setting
Basque	118.15401	BPE-MR
Basque	111.06085	BPE-V
Basque	92.91544	WP
Basque	79.4696	SPM
Basque	90.30664	Morfessor
English	118.04372	BPE-MR
English	104.06554	BPE-V
English	102.49587	WP
English	103.64609	SPM
English	99.97943	Morfessor
Finnish	118.35456	BPE-MR
Finnish	102.64364	BPE-V
Finnish	88.0279	WP
Finnish	107.97796	SPM
Finnish	89.03298	Morfessor
French	116.70251	BPE-MR
French	110.43207	BPE-V
French	111.92224	WP
French	103.60081	SPM
French	94.80364	Morfessor
German	115.13901	BPE-MR
German	90.91181	BPE-V
German	96.20926	WP
German	98.88095	SPM
German	88.12779	Morfessor
Greek	101.65912	BPE-MR
Greek	94.63509	BPE-V
Greek	83.97469	WP
Greek	75.78994	SPM
Greek	79.21414	Morfessor
Hebrew	97.93649	BPE-MR
Hebrew	111.71284	BPE-V
Hebrew	115.14652	WP
Hebrew	99.35821	SPM
Hebrew	99.93975	Morfessor
Hindi	121.32523	BPE-MR
Hindi	117.80531	BPE-V
Hindi	113.33367	WP
Hindi	104.81733	SPM
Hindi	111.70704	Morfessor
Indonesian	123.48862	BPE-MR
Indonesian	93.06506	BPE-V
Indonesian	78.16381	WP
Indonesian	97.51822	SPM
Indonesian	101.77236	Morfessor
Japanese	101.68905	BPE-MR
Japanese	92.05117	BPE-V
Japanese	103.28315	WP
Japanese	117.11949	SPM
Japanese	93.27034	Morfessor
Korean	100.53884	BPE-MR
Korean	90.70085	BPE-V
Korean	83.07006	WP
Korean	91.91427	SPM
Korean	70.47601	Morfessor
Mandarin	91.13514	BPE-MR
Mandarin	101.67698	BPE-V
Mandarin	98.01447	WP
Mandarin	86.54655	SPM
Mandarin	72.89142	Morfessor
Persian	126.21472	BPE-MR
Persian	124.11475	BPE-V
Persian	117.03348	WP
Persian	118.15876	SPM
Persian	120.27227	Morfessor
Russian	119.61481	BPE-MR
Russian	95.24603	BPE-V
Russian	100.80284	WP
Russian	81.85579	SPM
Russian	89.14557	Morfessor
Spanish	121.2689	BPE-MR
Spanish	102.31407	BPE-V
Spanish	100.99766	WP
Spanish	90.48757	SPM
Spanish	88.69318	Morfessor
Tagalog	115.89238	BPE-MR
Tagalog	99.35635	BPE-V
Tagalog	96.20716	WP
Tagalog	93.0288	SPM
Tagalog	81.66128	Morfessor
Thai	101.32569	BPE-MR
Thai	103.87144	BPE-V
Thai	104.93416	WP
Thai	102.25179	SPM
Thai	79.4985	Morfessor
Turkish	105.6798	BPE-MR
Turkish	97.74496	BPE-V
Turkish	103.16006	WP
Turkish	90.41201	SPM
Turkish	88.97014	Morfessor
Vietnamese	118.29179	BPE-MR
Vietnamese	116.66717	BPE-V
Vietnamese	110.26	WP
Vietnamese	108.47192	SPM
Vietnamese	94.61345	Morfessor

## Appendix E. SuE score: figures of results

See (figures 9 and 10).
